# Design and Fabrication of Strong Parts from Poly (Lactic Acid) with a Desktop 3D Printer: A Case with Interrupted Shell

**DOI:** 10.3390/polym11050760

**Published:** 2019-04-30

**Authors:** Vladimir E. Kuznetsov, Azamat G. Tavitov, Oleg D. Urzhumtsev, Mikhail V. Mikhalin, Alexey N. Solonin

**Affiliations:** Department of Physical Metallurgy of Non-Ferrous Metals, National University of Science and Technology “MISIS”, Leninskiy Prospekt 4, NUST MISIS, Moscow 119049, Russia; aztapps@gmail.com (A.G.T.); darikcr@gmail.com (O.D.U.); mih.mikhalin@ya.ru (M.V.M.); solonin@misis.ru (A.N.S.)

**Keywords:** design for additive manufacturing, desktop 3D printing, fused filament fabrication, fused deposition modeling, polylactic acid, mechanical strength

## Abstract

The ability to form closed cavities inside the part printed is an important feature of Fused Filament Fabrication technology. A typical part consists of a dense shell bearing the primary load, filled with low-density plastic scaffold (infill). Such a constitution of the part provides in most cases appropriate strength and low weight. However, if the printed part shape includes horizontal (orthogonal to printer’s Z axis) flat surfaces other than its top and bottom surface, then the shell of the part becomes interrupted, which may lead to drastic drop in the ability of the part to withstand loads. In the current study, a representative sample of a part with interrupted shell and testing apparatus is developed. Influence of shell and base thicknesses, as well as influence of the infill density on the part strength, are studied. Different approaches to the sample shape modification were applied and tested. The part shape optimization made with respect to peculiarities of Fused Filament Fabrication technology resulted in increment of the force, required to fracture the part from 483 to 1096 N and in decreased part mass from 36.9 to 30.2 g.

## 1. Introduction

Modern digital additive fabrication machines (3D printers) are often subdivided into “desktop” (personal), “professional,” and “industrial” ones. The main criterion determining whether the particular model belongs to one or another segment is the cost of the device. Thus, the desktop machines usually cost up to US$5000 (while most of them in this segment are below US$2000) [1]. The cost of most “professional” models is denoted with five digits, and the “industrial” machine rates are of six digits. The category of desktop additive machines features devices based on resin curing [2] and even on laser sintering [3], but the absolute majority of them are working on the principle of FDM [4,5,6] or FFF [7]. The broad spread of FFF technology happened in the last decade thanks to the RepRap [8,9,10] and other open projects.

Due to low cost of devices and consumables for them, the desktop FFF 3D printers are becoming the competitors for the traditional production processes. A small business with 10 to 30 3D printers can effectively compete in the market for fast production of small batches with companies using the technology of vacuum molding of plastics or RIM. Thousands of 3D printers owned by individual users can quickly accept the order to manufacture a batch of thousands of parts using platforms such as 3D hubs [11]. The potential of desktop (home, amateur, or personal) devices is huge. There are around 380 million tons of plastics of all kinds produced in the world annually [12], which means that about 50 kg of products made of plastic are consumed per one inhabitant of the planet. An average desktop device based on FFF technology is able to squeeze up to 5 mm^3^ of polymer every second through a nozzle. Although not capable to operate continuously in the 24/7 mode, each of the printers is, nevertheless, potentially capable of annual production with a total mass of at least 100 kg. That means that productivity of a *personal* 3D printer can fulfill all the needs in plastic products of a *person*. Although the Star Trek replicator [13,14] is not yet invented, desktop machines seem to be suitable for the role of functional prototypes of future distributed manufacturing cells [15,16,17,18,19,20,21,22,23,24]. These machines have found their application in different fields, from science [25,26,27,28,29] to toys [30] and from space [31,32] to farms [33,34]. However, until now, it is hard to say that desktop 3D printers were widely considered as production tools. The limited use of desktop printers for manufacturing of functional parts capable to handle significant loads is largely caused by lack of trust for the new fabrication means by designers and engineers. This lack of trust is backed by the widespread belief that the strength of 3D printed parts is dramatically lower in comparison with parts manufactured using traditional technologies, based on subtraction or deformation of material. Understanding the peculiarities of 3D printing technology and designing parts while keeping these peculiarities in mind allows, at least, an approaching of the strength of monolithic polymer parts. There is a significant amount of attention drawn to develop design rules for additive manufacturing [35,36] in order to achieve desired geometry. A certain amount has been drawn to determine optimal materials and printing modes [37]. There are also studies on the influence of parts topology on mechanical properties of FFF printed parts [38,39]. However, there is a shortage in studies connecting 3D printed parts properties to their shape and constitution.

**Figure 1:**
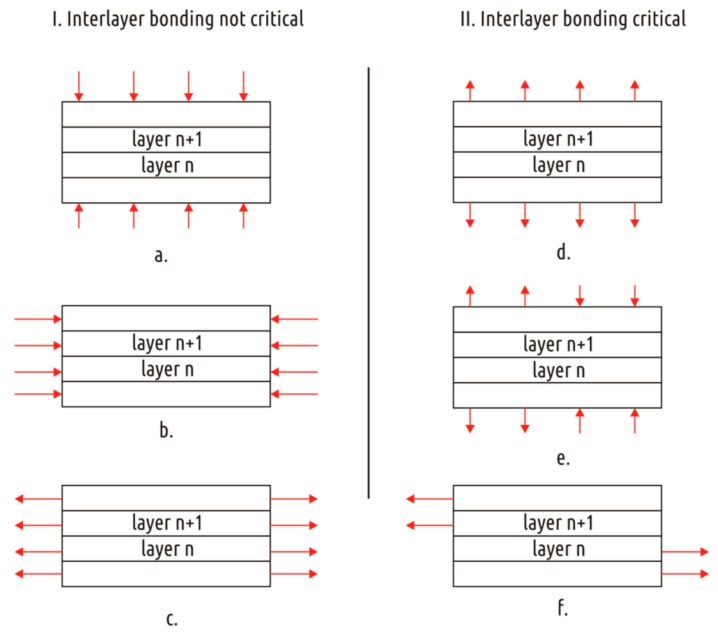
Two categories of stressed layered parts. Their interlayer bonding is not (**I.**) and is (**II.**) critical. The category I. includes cases of compression along the Z axis with no buckling (**a**) and compression (**b**) and tension (**c**) orthogonal to Z axis. The category II. includes cases of tension (**d**) and bending (**e**) along Z axis and torque or shear orthogonal to the Z axis (**f**).

With all the variety of parts, the operation of which implies bearing mechanical loads and the manufacturing method is FFF 3D printing, they can all be divided into two categories (Figure 1) depending on stress state w.r.t. layer boundaries. It is assumed here and below that coordinate axes of the part are equal to the axes of the printer. The threads forming the part lie in the planes parallel to XY axes, and thus, orthogonal to the Z axis. The purpose, shape, and printing orientation for the parts of the first category are such that the bonding strength between the layers does not play the critical role. In the part of the second category the weak spot, on the contrary, is situated on the layer boundary and the cohesion between the layers determines the strength of the whole part.

The first category includes parts that are compressed along Z axis during operation and that do not experience risk of buckling (Figure 1a), for example, the “amplifier flat stand” [40]. The first category also includes parts printed in such a way that critical stresses are applied along the layers (in the XY plane) and the stress state is almost independent from the Z axis coordinate (Figure 1b,c), for example, “Knuckle Duster” [41] or “Bag Holder” [42]. There are no questions on how to align such flat parts on the printer bed: The most stable position is the most appropriate regarding the stress distribution. Another type of models can be exemplified by “Fixing Angle” [43]. They fall into this category only if correctly oriented w.r.t the printer bed. The behavior of such parts under load will differ little from the behavior of parts of the same shape and material obtained by traditional methods, such as injection molding. Accordingly, shape optimization for such parts should use the accumulated experience in designing parts for traditional production methods.

The second category comprises parts that stretch or bend along or twist around the Z-axis during operation (Figure 1d–f). Such parts experience normal tensile stresses or tangential shear stresses acting on the layer’s boundaries. With structural features of FFF printed parts taken into account, it makes sense to subdivide the second category into two.

The first subcategory includes parts with the shell not being interrupted during the printing process: polymer threads forming the shell of the next layer lie completely or partially on the threads forming the shell of a previous layer. An example of such part is the “hammer” [44] or the “box spanner adapter” [45]. The strength of parts from this subcategory is determined primarily by the strength of its shell. Accordingly, a simple and efficient recipe for increasing the strength of such parts is to invest material and printing time into shell formation. Ways to increase the shell strength (layer cohesion) for PLA FFF parts are studied in References [46,47].

**Figure 2:**
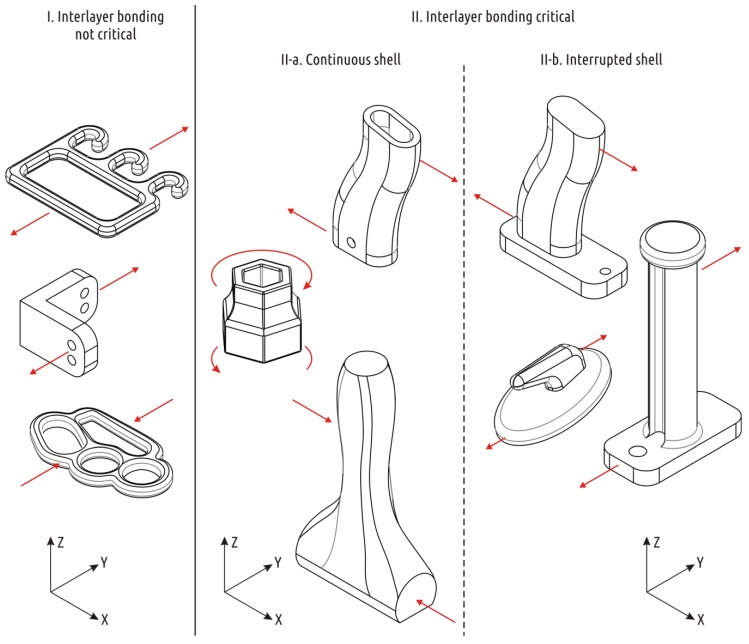
Examples of 3D printed parts experiencing significant stress during operations.

Finally, the second subcategory comprises parts which have a shell that is interrupted one or more times during the printing process, that is, there are times when the threads forming the shell of the next layer lie on the filaments forming the base, infill, or support. Such models can be exemplified by the “Spool holder” [48], “Plane Handle” [49], or the “Hook” [50]. These parts have flat faces parallel to the XY plane outside the upper and lower bases of the part. Examples of parts of all the categories and subcategories considered are shown in Figure 2.

Behavior of critically stressed parts with shell interruptions (II-b.) seems to be the least predictable in comparison with others (I and II-a.). The hypothesis is that the interruption of shell in the most stressed area of a loaded 3D printed part will result in unacceptably low mechanical performance. Thus, the current work is devoted to the study of parts with interrupted shells and the ways to increase the strength of such parts by modifying its shape with respect to its constitution. 

## 2. Methods and Materials

### 2.1. Sample Shapes

**Figure 3:**
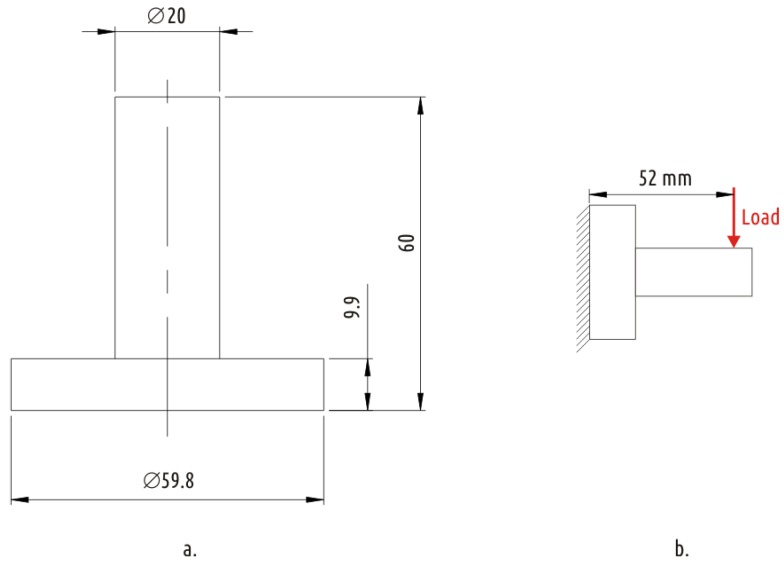
Basic sample dimensions (**a**) and loading (**b**) scenario.

An item consisting of two connected coaxial cylinders of large (boss) and small (shaft) diameters was selected as a representative of part with an interrupted shell (Figure 3a). As a test procedure, radial load is applied to the shaft with the boss is rigidly fixed (Figure 3b). Obviously, the most loaded (and the weakest) area of the part is the junction between the shaft and the boss, where the shell is interrupted.

**Figure 4:**
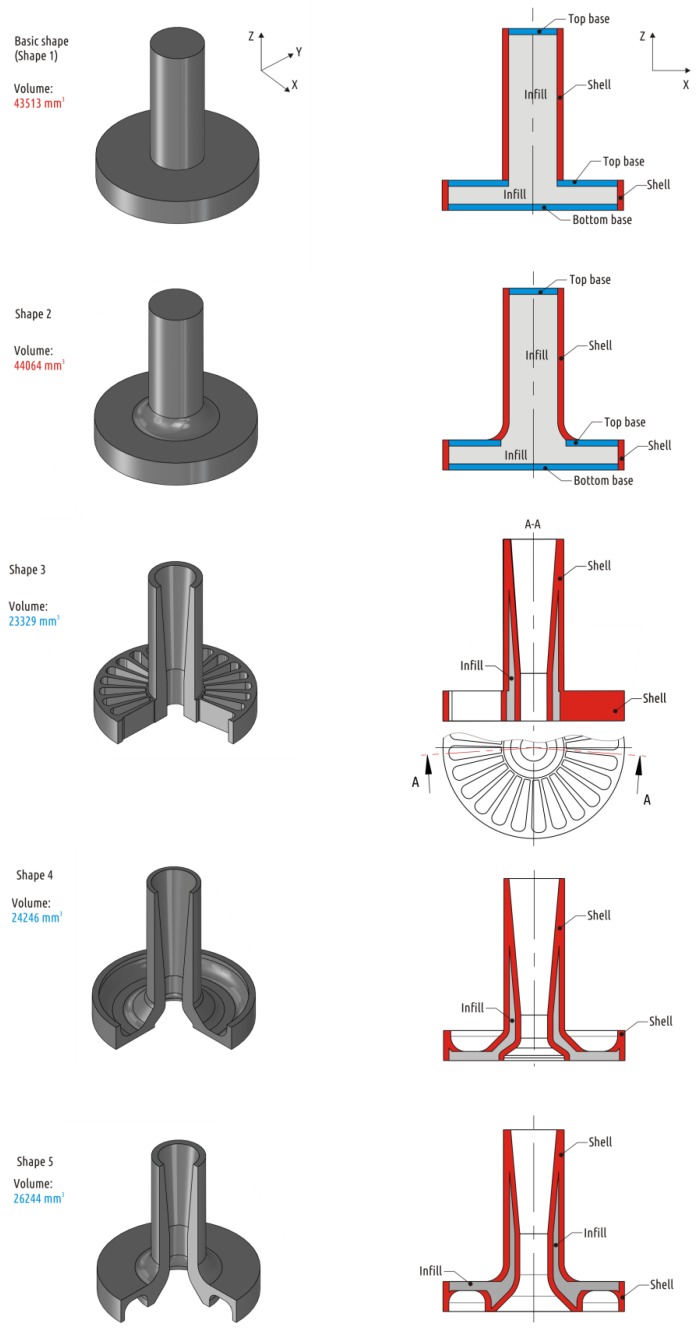
Part shapes studied: CAD models (**left**) and constitution of 3D printed samples (**right**).

Based on the shape described above and keeping main dimensions intact, four extra CAD models were prepared and tested in attempts to improve the part mechanical performance (Figure 4).

Shape 2 represents traditional approach to improve loaded part geometry—adding a fillet to the critical corner. The fillet literally rounds the sharp transition, removing the apparent stress concentrator and also adds material to the most loaded area. Adding a fillet with a radius of 6 mm significantly increases the interface area between the shaft and the boss.

**Figure 5:**
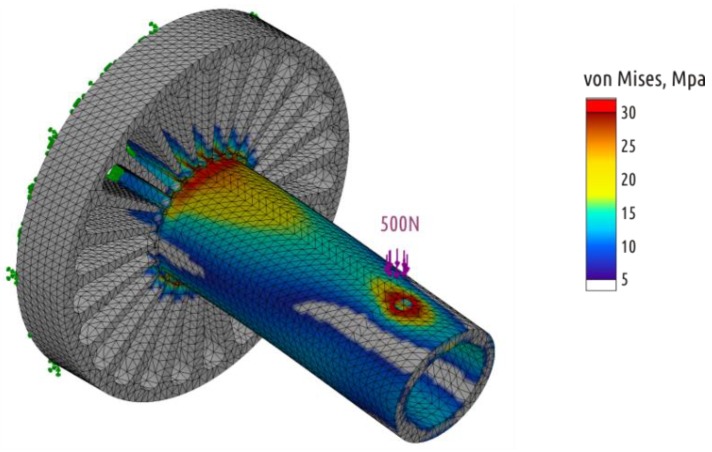
Stress distribution in the Shape 3 (SolidWorks Simulations).

Shape 3 represents an intuitive attempt to solve the interrupted shell issue made with respect to 3D printed part constitution (the superposition of threads mimicking shells, bases, and infills). When working with traditional manufacturing methods, whether it is molding, casting, forming, or machining, it is impossible to make the part stronger by removing some of the CAD model volume. In the case of the FFF technology, removing some of the volume from the CAD model does not necessarily imply reduction of the physical product mass. Additional open or closed cavities in the model lead to the appearance of additional shells in the slicer (and in the part printed). Shape 3 external dimensions are preserved same to the Shape 1, but axial and radial cuttings are added. As a result, thread deposition paths are generated completely differently: The shaft shell is now passing through the boss and is printed from the very printer table. In fact, the shell becomes continuous. Since the Shape 3 sample is formed exclusively from the shell and 100% infill, it can be considered a solid body and analyzed with computer-aided simulation techniques. An example of adequate numeric simulation of loaded 3D printed PLA part can be found in References [37,51]. In current paper, the SolidWorks Simulations extension of SolidWorks 2017 was used. The Figure 5 shows the stress distribution in the loaded areas of Shape 3. 

**Figure 6:**
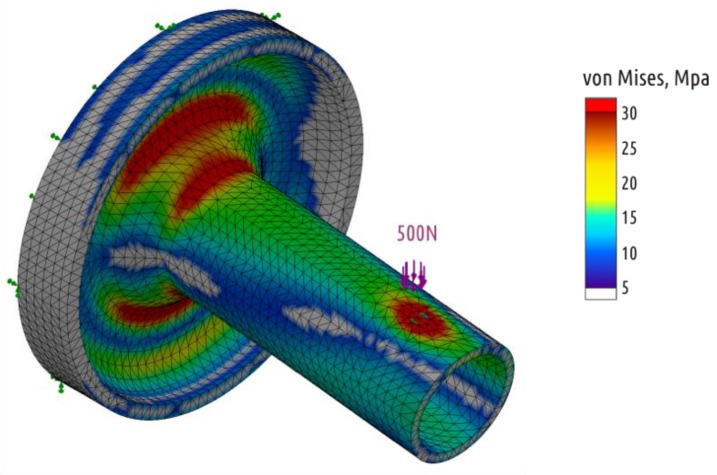
Stress distribution in the Shape 4 (SolidWorks Simulations).

After several cycles of shape modification and stress analysis, Shape 4 was designed, where the calculated stresses are distributed between the shaft and the base (Figure 6). At the same time, it fully fits into the volume of the basic shape. 

Due to the existence of a through hole along the shaft axis, there are two shells (like in Shape 3) instead of one in it (like it was in Shapes 1 and 2), the inner and the outer ones. The inner shell of the Shape 4 part is continuous and the outer shell is interrupted, but it stands on the 100% infill foundation.

Finally, Shape 5 represents combined approach, with modification of initial part both by adding and subtracting CAD model volume. Shape 5 was obtained by removing the volume from the least loaded sections of Shape 2. The fillet (added volume) provides graduate transition from shaft to boss, while the axial cutting leads to forming of an extra continuous shell. 

For CAD models with relatively large volumes (Shape 1 and Shape 2), eight different configurations were tested. Three parameters describing 3D printed part constitution varied at two levels: Shell thickness (1.2 and 2.4 mm), base thickness (0.6 and 1.2 mm), and infill value (20 and 60%). For CAD models with lower volume a single configuration was tested: Shell thickness 1.8 mm, 100% infill, and no bases (base thickness 0 mm).

### 2.2. Samples Fabrication

A desktop Ultimaker 2 (Ultimaker B.V., Geldermalsen, Netherlands) printer was used to produce all the samples. The specific machine used differs from the mass-market model with an installed alternative feed mechanism of BondTech (Bondtech AB, Värnamo, Sweden) brand, built on a stepper motor with an integrated gearbox and drive to both feed rollers, and an alternative 3D Solex (Cepta AS, Oslo, Norway) heating unit with an increased power heating element (~50 W). The alternative heating unit, unlike the stock one, allows changing nozzles. In this series of experiments, a brass nozzle with a channel diameter of 0.6 mm was used instead of the 0.4 mm standard nozzle in stock Ultimaker 2 hot end.

The poly (lactic acid), or PLA, is used as the material for samples fabrication. The main advantage of PLA in comparison to other polymers and blends used for FFF 3D printing is the low level of shrinkage and relatively low melting temperature. Other advantages of PLA include its biodegradability, absence of unpleasant odors when heated, and its overall environmental compatibility in all aspects of the life cycle. PLA emits ten times less potentially dangerous ultra-fine particles [52] than ABS and can withstand at least 25 kiloGray of gamma irradiation with no degradation of mechanical properties [53]. The most important disadvantages of the PLA are its relatively low softening temperature (the Vicat point is 55 °C), which makes it incompatible with elevated temperature environment, and deterioration of mechanical properties caused by hydrolysis [54], which makes it incompatible with wet environments. A turquoise PLA filament was used, produced by REC Company (Moscow, Russia). All the material came from the same batch produced in June 2018, according to the labels (six months before the experiment). The claimed diameter of the filament was 2.85 mm, but the actual average diameter, calculated on 60 measurements of six different spools, was 2.83 mm with standard deviation of 0.02 mm. This specific manufacturer of filament was chosen due to locally produced material and the desire to obtain results comparable with previous studies [46,47]. Papers [46,55] show that all other characteristics being identical the filament color influences strength of the products made from it. Thus, filament of the same color was used as in previous studies.

The values of the printing parameters remained constant during all experiments:

- extrusion temperature (210 °C);
- nozzle diameter (0.6 mm);
- heated bed temperature (60 °C);
- first and subsequent layer thickness (0.3 mm);
- first layer printing speed (25 mm/s);
- subsequent layers printing speed (30 mm/s);
- flow rate (5.4 mm^3^/s).

For each observation mentioned in the work, a lot of five samples was made and tested. The paper presents the average values for each test lot, while the standard deviation is indicated after the average value in brackets. 

The sample was placed at the center of the printer bed. The G-code file was prepared using Cura 15.02.1 software (slicer). 

All samples were weighed before mechanical testing using digital analytical scales ViBRA LF Series (Shinko Denshi Co. LTD, Tokyo, Japan). Measurement results were rounded to one decimal digit. 

### 2.3. Mechanical Testing

**Figure 7:**
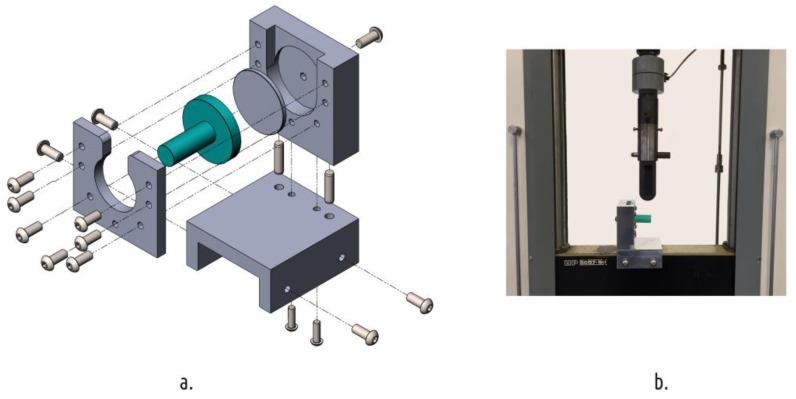
Sample fixture (**a**) installed on the universal testing machine (**b**).

Sample strength tests were carried out on a standard universal electromechanical testing machine IR 5057-50 (OOO Tochpribor, Ivanovo, Russia) with a digital control system. The samples were fixed with a specially designed and manufactured device (Figure 7). That fixture was mounted on a movable traverse of the testing machine. The top roller from the three-point bend test kit was used to apply load on the sample shaft. 

The tests were carried out at constant speed (10 mm/min) and were held on until the sample was destroyed. During the tests displacements and loads were recorded. The reference point was the state of the machine with a load of 5N applied to eliminate mounting clearances.

The part strength was assumed to be equal to the fracture load (load at which the first apparent crack appears). Along with absolute strength, the relative strength (fracture load related to the sample mass) was also considered.

## 3. Results and Discussion

### 3.1. Basic Shape

The test results for eight configurations of Shape 1 are shown in the Table 1.

**Figure 8:**
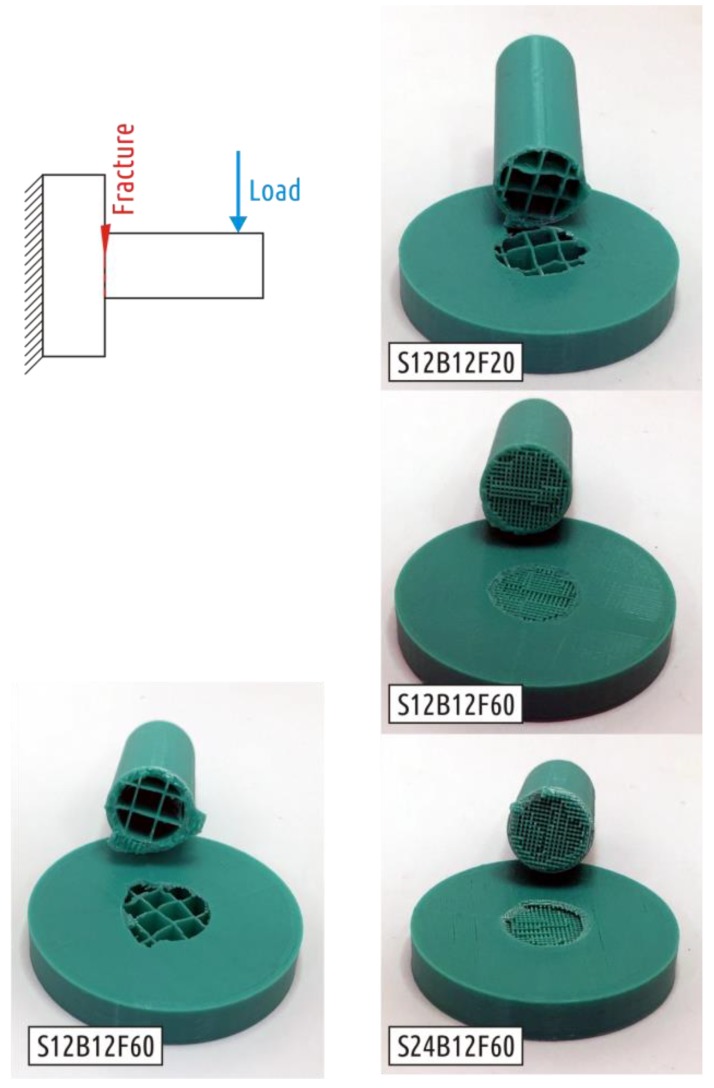
Fracture of Shape 1 samples.

**Figure A1:**
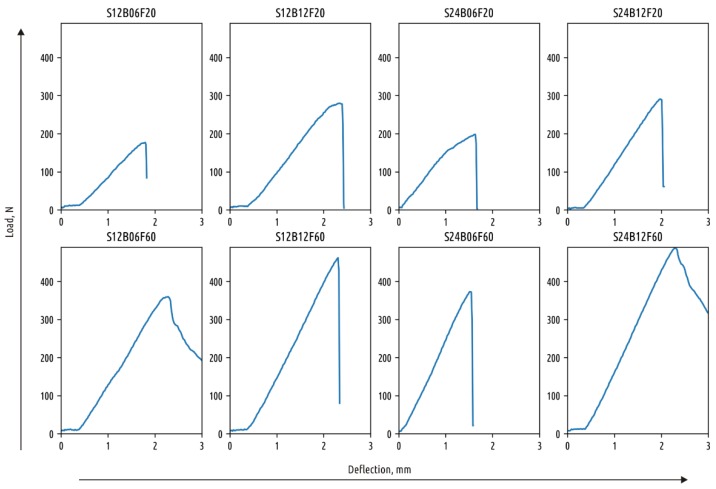
Typical loading curves for Shape 1 samples manufactured in different configurations.

The loading curves of the characteristic representatives for each of the lots tested are shown in the Appendix A, Figure A1. In general, the strength of all the considered configurations is very modest. The shaft itself is quite durable, but it becomes easily separated from the boss (all of the samples examined were destroyed at the interface between the shaft and the boss, see Figure 8. The reason for this lies in the fact that the strong shaft stands on the loose base of the thread grid forming the boss infill. The connection between the shaft and the boss passes through the infill and along the boundary between the upper base of the boss and the shaft shell. In other words, the part becomes weak due to the shell interruption.

**Figure 9:**
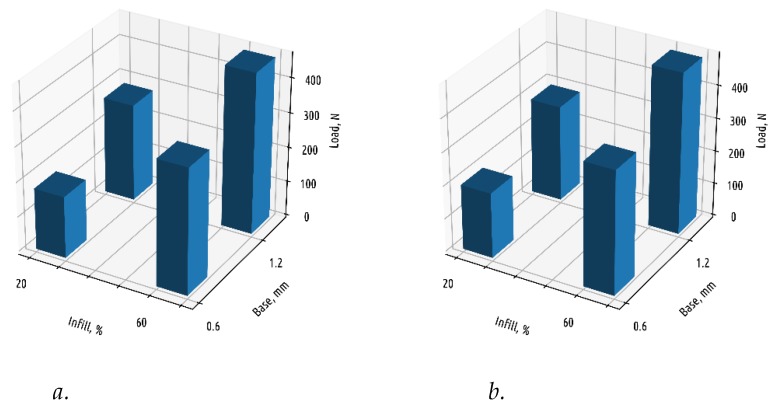
Fracture load depending on base thickness and infill density for samples of Shape 1 with shell thickness equal to 1.2 (**a**) and 2.4 (**b**) mm.

As it can be seen from Figure 9, increasing the base thickness and infill percentage has noticeable effect on the part strength, while the shell thickness has the minimal influence. Thus, the rule that works well for parts with a continuous shell (in order to increase the part strength, it is necessary first of all to invest time and material into shell) is absolutely inapplicable to parts of the shape considered.

Acceptable values of part strength can be achieved by further increasing the infill value (up to 100%), but this will obviously lead to significant increase in the part mass. Considering relative values, one can see that increasing infill density from 20 to 60% leads to a negligible increase in relative strength. It is more rational to modify the part shape while keeping the main (coupling) dimensions intact. 

### 3.2. Shape Modification—The Traditional Approach (Shape 2)

**Figure A2:**
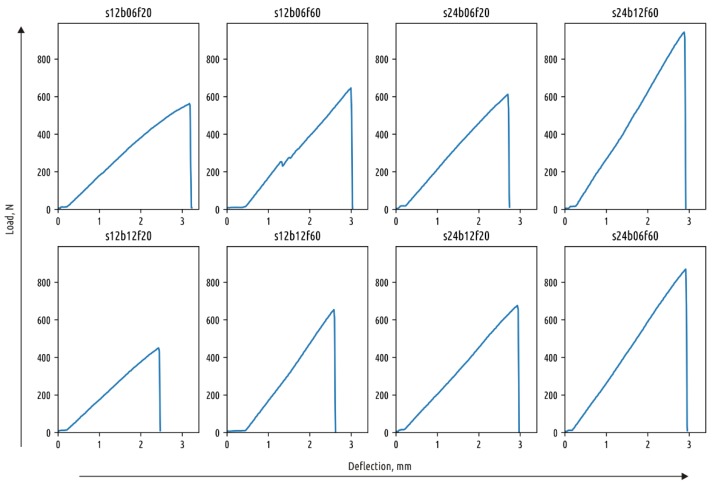
Typical loading curves for Shape 2 samples manufactured in different configurations.

The test results for eight configurations of Shape 2 are shown in Table 2, and characteristic loading curves are presented in the Appendix A, Figure A2. 

**Figure 10:**
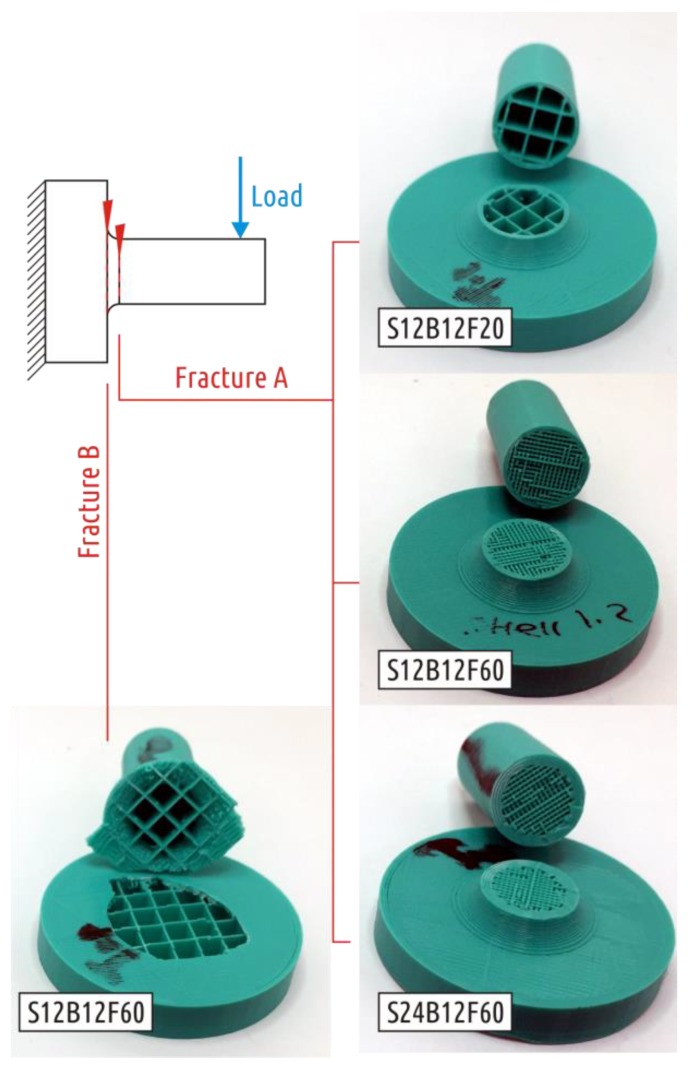
Two types of Shape 2 samples fracture.

As can be seen from the results, simply adding a fillet dramatically affects the strength of the part. The smallest recorded strength for Shape 2 samples exceeds the maximum recorded one for Shape 1. Moreover, the nature of parameter influence changes completely. Samples with thicker shells (2.4 mm) and low infill density (20%) break at the interface between the boss and fillet. All others fail at the boundary between the fillet and the cylindrical part of the shaft (Figure 10). 

**Figure 11:**
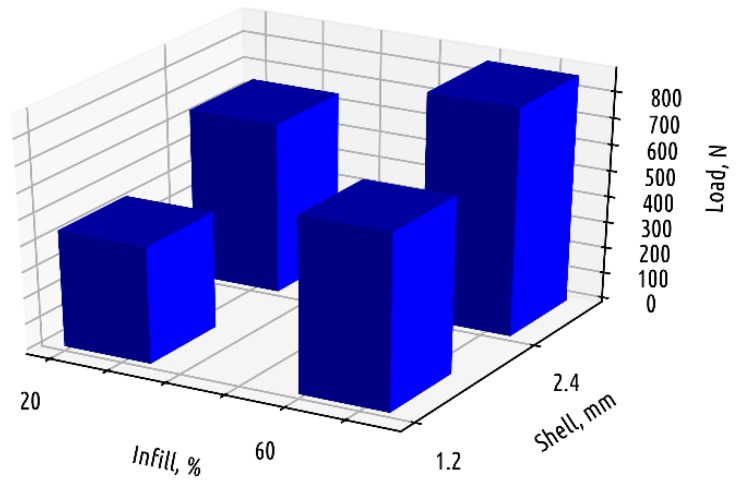
Fracture load depending on base thickness and infill density for samples of Shape 2.

Accordingly, in most cases the base thickness does not have any influence, since the material forming the base does not lie in the critical zone. The difference in results between samples that only vary in base thickness is statistically insignificant. Thus, the number of configurations considered for Shape 2 samples can be reduced from eight to four (Figure 11).

The infill density has a great influence on the absolute part strength for Shape 2, but for the relative strength, shell thickness becomes paramount: increase in the infill rate from 20 to 60% with other things remaining unchanged leads to a decrease in the relative part strength. 

### 3.3. Modifying the Shape with FFF Technology Specificity in Mind (Shape 3)

**Figure 12:**
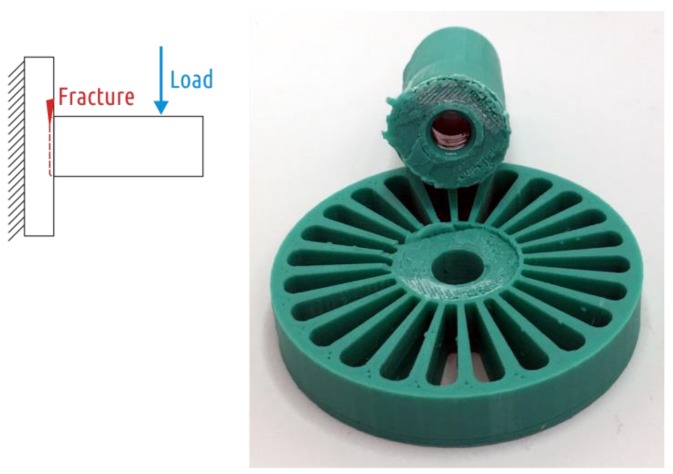
Fracture of Shape 3 samples.

**Figure A3:**
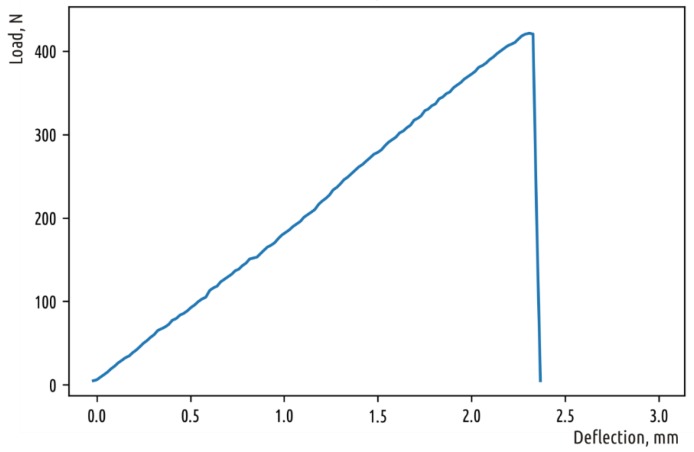
Typical loading curve of Shape 3 samples.

The characteristic test curve for the Shape 3 sample is shown in the Appendix A, Figure A3. Sample destruction occurred over the shaft section, slightly submerged (1–3 mm) into the boss (Figure 12).

The average absolute strength of Shape 3 samples was 426 (18) N with a mass of 27.0 (0.1) g. The relative strength of the part was accordingly 15.8 N/g. The results obtained are inferior to the best absolute records obtained for Shape 1, but exceed the best relative ones. The results are significantly inferior to those obtained for Shape 2. It is important to note that Shape 3 fits into the basic shape volume, while Shape 2 has an element (fillet) protruding beyond its dimensions.

### 3.4. Shape Optimization Using CAE (Shape 4)

**Figure 13:**
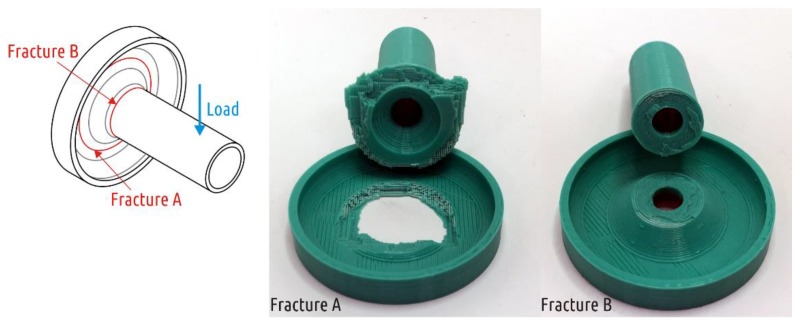
Two kinds of Shape 4 fracture.

**Figure A4:**
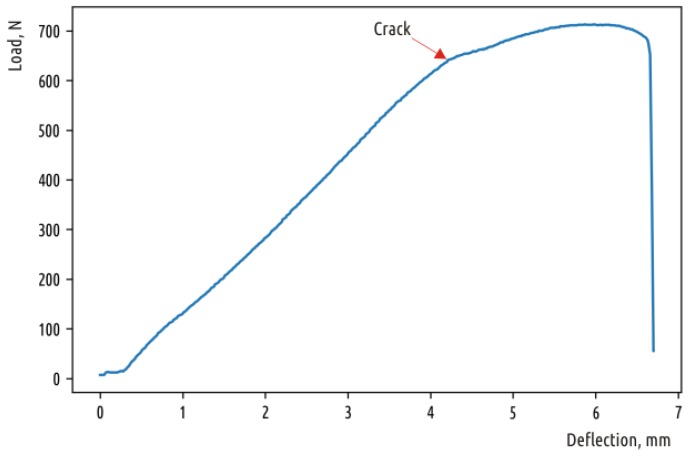
Typical loading curve for Shape 4.

In all previous cases, the load at which crack occurred was the largest load on testing curve. Four of five Shape 4 samples tested exhibited another behavior under critical loads (Appendix A, Figure A4). After the appearance of the first crack, the load required for further deformation continues to increase. That is, the appearance and growth of a crack does not immediately lead to the shaft separation from the boss, and the crack appears and grows in the boss part of the sample (Figure 13, fracture A). One of five specimens tested fractured at the point of transition of the cylindrical shaft into a conical inlet (Figure 13, fracture B). If we come back to stress distribution in Figure 6, it can be seen that computer simulation revealed two critical zones. As the experiment showed, the sample destruction is possible in each of them with different probability. 

The more likely fracture (Figure 13, fracture A) passes through the entire boss that is formed by 100% infill. Due to the mutually orthogonal threads arrangement of different infill layers, the crack in the sample does not only grow along the borders between the individual plastic threads, but also across the threads. It is the latter phenomenon that ensures the ductile nature of sample destruction. 

If the Shape 4 part strength is to be defined as the load corresponding to the crack appearance, the average strength is 662 (51) N with a mass of 27.6 (0.1) g. Accordingly, the relative strength of the part was 24.0 N/g.

### 3.5. Combining Approaches (Shape 5)

**Figure 14:**
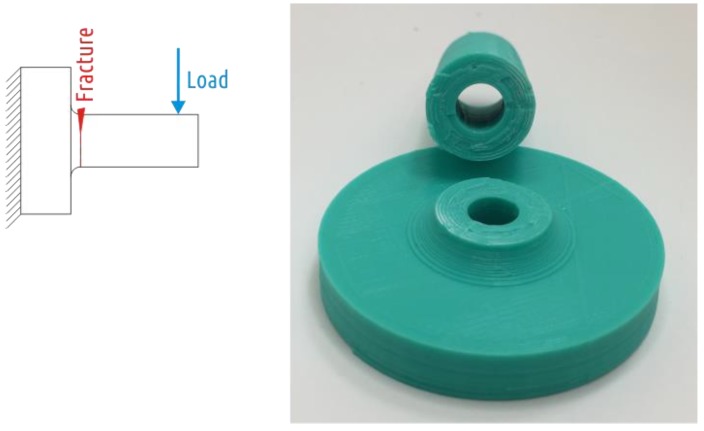
Shape 5 samples fracture.

**Figure A5:**
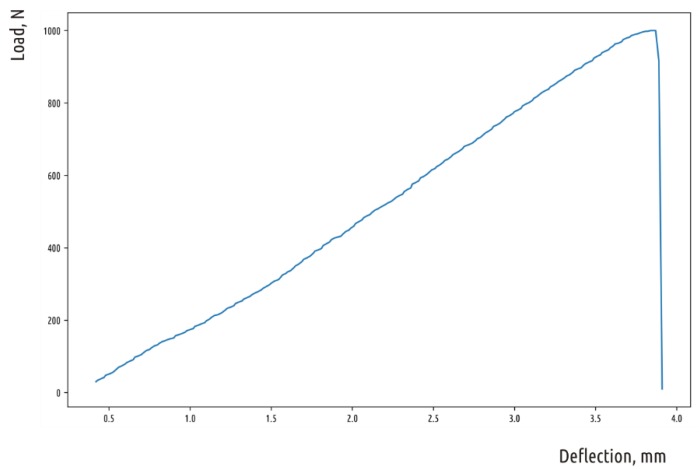
Typical loading curve for Shape 5.

The fracture of all Shape 5 samples occurred where the cylindrical shaft transitions into the fillet (Figure 14), the characteristic test curve is shown in the Appendix A, Figure A5. 

The average strength values for the Shape 5 samples amounted 1096 (72) N with a mass of 30.3 (0.2) g. The relative strength of the sample was 36.2 N/g. 

### 3.6. Summary

**Figure 15:**
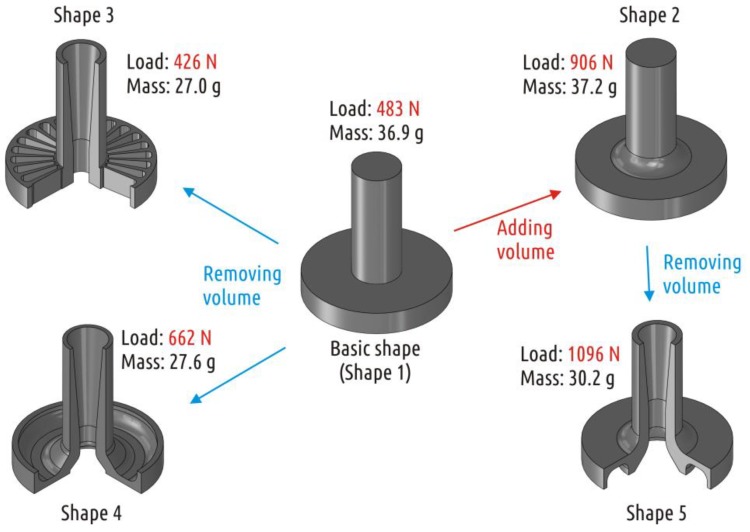
Shape optimization path and results.

A summary of results is shown in Figure 15. Shape 1 can be considered an example of poor design: the sharp transition from the boss to the shaft is a flaw even for traditionally manufactured parts. In case of 3D printing, such a transition implies shell interruption and critical weak spot appearance. As it is shown by results for shapes 3 and 4, redistribution of material within a given, initially flawed, shape can significantly increase the part strength, but the geometry optimization effect taking into account 3D printing features is less significant than the effect of geometry modification performed in accordance with the basic principles of designing products for convenient manufacturing (samples of Shape 2). The maximum effect is achieved by combined approach (Shape 5).

As a result of the shape modification without going beyond the initial geometric boundaries, it was possible to increase the strength (relative strength):

- for the model with a sharp transition between the boss and the shaft (Shape 1 to Shape 4) by 1.37 times (1.83 times);
- for the model with a smooth transition between the boss and the shaft (Shape 2 to Shape 5) by 1.13 times (1.40 times).

Additional reserves to improve the part strength for an optimized shape can be sought in technological parameter optimization of the printing process. 

## 4. Conclusions

In order to increase FFF part strength, its geometry can be optimized both by adding volume to the CAD model (rounding, adding fillets, smooth transitions) and by introducing cavities, and thus, by providing extra shells in the critical sections and by converting interrupted shells into continuous ones. This technique of shape optimization for the FFF production technology differs from that one for traditional production methods (such as casting, forming, machining), as well as other digital additive technologies (SLA, SLS, LOM), where extra cavities would not contribute to the absolute strength of a part. 

Computer simulation methods are applicable to analyze the behavior of FFF part models under load if they are printed with infill only with 100% density (or without infill invocation), at least at a qualitative level.

## Figures and Tables

**Table 1:** Test results for Shape 1 configurations.

Lot#	Code	Shell, mm	Base, mm	Infill, %	Sample Mass, g *	Fracture Load, N	Relative Strength, N/g
1	S12B06F20 **	1.2	0.6	20	17.6 (0.1)	187 (13)	10.6
2	S12B06F60	1.2	0.6	60	33.4 (0.2)	365 (24)	10.9
3	S12B12F20	1.2	1.2	20	21.0 (0.1)	275 (18)	13.1
4	S12B12F60	1.2	1.2	60	34.9 (0.2)	462 (24)	13.2
5	S24B06F20	2.4	0.6	20	22.1 (0.1)	201 (28)	9.1
6	S24B06F60	2.4	0.6	60	35.6 (0.1)	357 (23)	10.0
7	S24B12F20	2.4	1.2	20	24.8 (0.1)	290 (28)	11.7
8	S24B12F60	2.4	1.2	60	36.9 (0.2)	483 (27)	13.1

*–hereinafter, the values in brackets are standard deviations; **–hereinafter, S—shell, B—base, F—infill.

**Table 2:** Test results for Shape 2 configurations.

Lot	Code	Shell, mm	Base, mm	Infill, %	Mass, g	Fracture Load, N	Relative Strength, N/g
1	S12B06F20	1.2	0.6	20	20.0 (0.1)	508 (30)	25.4
2	S12B06F60	1.2	0.6	60	34.1 (0.2)	648 (12)	19.0
3	S12B12F20	1.2	1.2	20	21.3 (0.2)	506 (36)	23.8
4	S12B12F60	1.2	1.2	60	35.4 (0.2)	650 (28)	18.4
5	S24B06F20	2.4	0.6	20	23.6 (0.1)	620 (36)	26.3
6	S24B06F60	2.4	0.6	60	35.9 (0.2)	896 (38)	25.0
7	S24B12F20	2.4	1.2	20	24.9 (0.2)	644 (45)	25.9
8	S24B12F60	2.4	1.2	60	37.2 (0.2)	906 (52)	24.4

## Abbreviations

In the current work, the following notions are introduced and the following shorthand is used:

*FDM*	Fused Deposition Modeling is a technology of digital additive manufacturing based on layered deposition of melted thermoplastic;
*FFF*	Fused Filament Fabrication, which is the same as FDM, with the only difference being that the *FDM* is a trademark of Stratasys Inc., while the *FFF* is a term coined inside the RepRap community. Since the current study involves open-source 3D printer, the term *FFF* is used;
*Filament*	Plastic in the filament form used as supply in *FFF* process;
*Thread*	Extruded and deposited thread of plastic mimicking the FFF part;
*Shell*	A component of FFF part, reproducing the lateral surface of 3D model. The shell of a layer consists of single or multiple equidistant perimeters formed by the thread. The number of perimeters and the thread wideness are responsible for the shell thickness;
*Infill*	An internal component of FFF part, formed by threads in orthogonal straight lines. The distance between the threads defines the infill density;
*Base*	A component of FFF part, reproducing flat surfaces parallel to the 3D printer bed. Base may consist of multiple layers, usually printed mutually orthogonally in XY directions, and can be disabled;
*Fracture load*	The load observed during mechanical testing at which the sample exhibits the first crack, in the context of the study, the fracture load is the sample strength;
*Relative Strength*	A ratio of sample *fracture load* to its mass;
*Flow rate* (mm^3^/s)	The volume of plastic delivered through the nozzle per unit time;
*Printing speed* or *Feed rate*	The speed of nozzle traveling across XY plane while extruding the plastic.
